# Suvorexant and mirtazapine improve chronic pain-related changes in parameters of sleep and voluntary physical performance in mice with sciatic nerve ligation

**DOI:** 10.1371/journal.pone.0264386

**Published:** 2022-02-25

**Authors:** Hisakatsu Ito, Hiroshi Tsuneki, Toshiyasu Sasaoka, Naoki Toyooka, Mitsuhiro Matsuo, Mitsuaki Yamazaki

**Affiliations:** 1 Department of Anesthesiology, University of Toyama, Toyama, Japan; 2 Department of Clinical Pharmacology, University of Toyama, Toyama, Japan; 3 Faculty of Engineering, University of Toyama, Toyama, Japan; Belgrade University Faculty of Medicine, SERBIA

## Abstract

Both chronic pain and sleep disorders are associated with a reduction in the quality of life. They can be both a cause and a consequence of each other, and should therefore be simultaneously treated. However, optimal treatments for chronic pain-related sleep disorders are not well established. Here, we aimed to investigate the effects of suvorexant, a novel sleep drug, and mirtazapine, a noradrenergic and specific serotonergic antidepressant, on pain-related changes in sleep parameters in a preclinical chronic pain mice model, by partial sciatic nerve ligation. We evaluated the quantity, duration, and depth of sleep by analyzing the electroencephalogram and voluntary activity by counting the number of wheel rotations to determine various symptoms of sleep disorders, including reduced total sleep time, fragmentation, low quality, and impaired activity in the daytime. Suvorexant and mirtazapine normalized the reduction in sleep time and fragmented sleep, further regaining the sleep depth at sleep onset in the chronic pain state in nerve-ligated mice. Mirtazapine also increased the percentage of rapid eye movement sleep in mice. Suvorexant decreased voluntary activity, which was prolonged after administration; however, mirtazapine did not decrease it. Although the effects of suvorexant and mirtazapine on sleep and activity are different, both suvorexant and mirtazapine could be potential therapeutic agents for chronic pain-related sleep disorders.

## Introduction

Chronic pain is pain that persists past normal healing time and is usually regarded as chronic when it lasts for more than 3 to 6 months [1]. Chronic pain often leads to further psychiatric disorders such as anxiety and depression, which significantly affect the health-related quality of life [2–4]. Sleep disorders are the most common psychiatric disorders related to pain [5–8], and sleep disorders exacerbate pain [9–12]. Sleep disorders and pain cause a vicious spiral and should be treated simultaneously in clinical situations. Common sleep drugs, benzodiazepines, and nonbenzodiazepines (also referred to as Z-drugs) have a narrow role in managing secondary sleep disorders caused by other mental or physical conditions, including pain [13]. Furthermore, the indications for these drugs should be limited due to their harmful side effects, such as falling [14], cognitive impairment [15], dependence [16], and increased risk of death [17]. Therefore, the effectiveness of novel drugs with targets other than GABA_A_ receptor including suvorexant and mirtazapine, for the treatment of insomnia disorders have attracted attention [18]. Further research is needed to develop new treatments for pain-related sleep disorders by using these novel drugs.

Hyperactivity of ascending monoaminergic neurons from the central arousal system to the brainstem could be one of the causes of pain-related sleep disorders [19, 20]. Suvorexant, a novel sleep drug, promotes a normal sleep state by blocking orexin receptors, which activate monoaminergic and cholinergic neurons in the central arousal system [21–24]. Mirtazapine belongs to the class of noradrenergic and specific serotonergic antidepressants (NaSSAs) [25]; however, it also suppresses histaminergic and serotonergic neurons and causes sleepiness, and is often used for the off-label treatment of insomnia [25–27]. Because of their pharmacological mechanisms, these drugs could be potential therapeutic agents for pain-related sleep disorders. Here, we performed a pre-clinical study on the effects of suvorexant and mirtazapine on sleep in a neuropathic pain model produced by partial sciatic nerve ligation (PSNL). We evaluated the total sleep time, percentage of REM sleep, and duration of each parameter of wakefulness, non-REM sleep, and REM sleep. Furthermore, we investigated the power density of delta waves during non-REM sleep to evaluate the depth of sleep.

## Materials and methods

### Animals

This study was carried out in accordance with the ARRIVE guidelines, the Act on Welfare and Management of Animals, and the recommendations in the Guidelines for Proper Conduct of Animal Experiments of the Science Council of Japan. The present study was conducted in accordance with the Guiding Principles for the Care and Use of Laboratory Animals at the University of Toyama, as adopted by the Committee of Ethics in Animal Experiments of the University of Toyama (Protocol Number: A2016UH-5). Male C57BL/6J mice, the most common animal line for pain research [28], were purchased from Japan SLC. The mice were maintained at 22–26°C with a 12-h light-dark cycle. Zeitgeber time (ZT) 0 and ZT 12 represent the light onset (07:00) and offset times (19:00), respectively. Food and water were provided *ad libitum*. Every effort was made to minimize the number and suffering of the animals used in the following experiments.

### Study design

**Fig 1:**
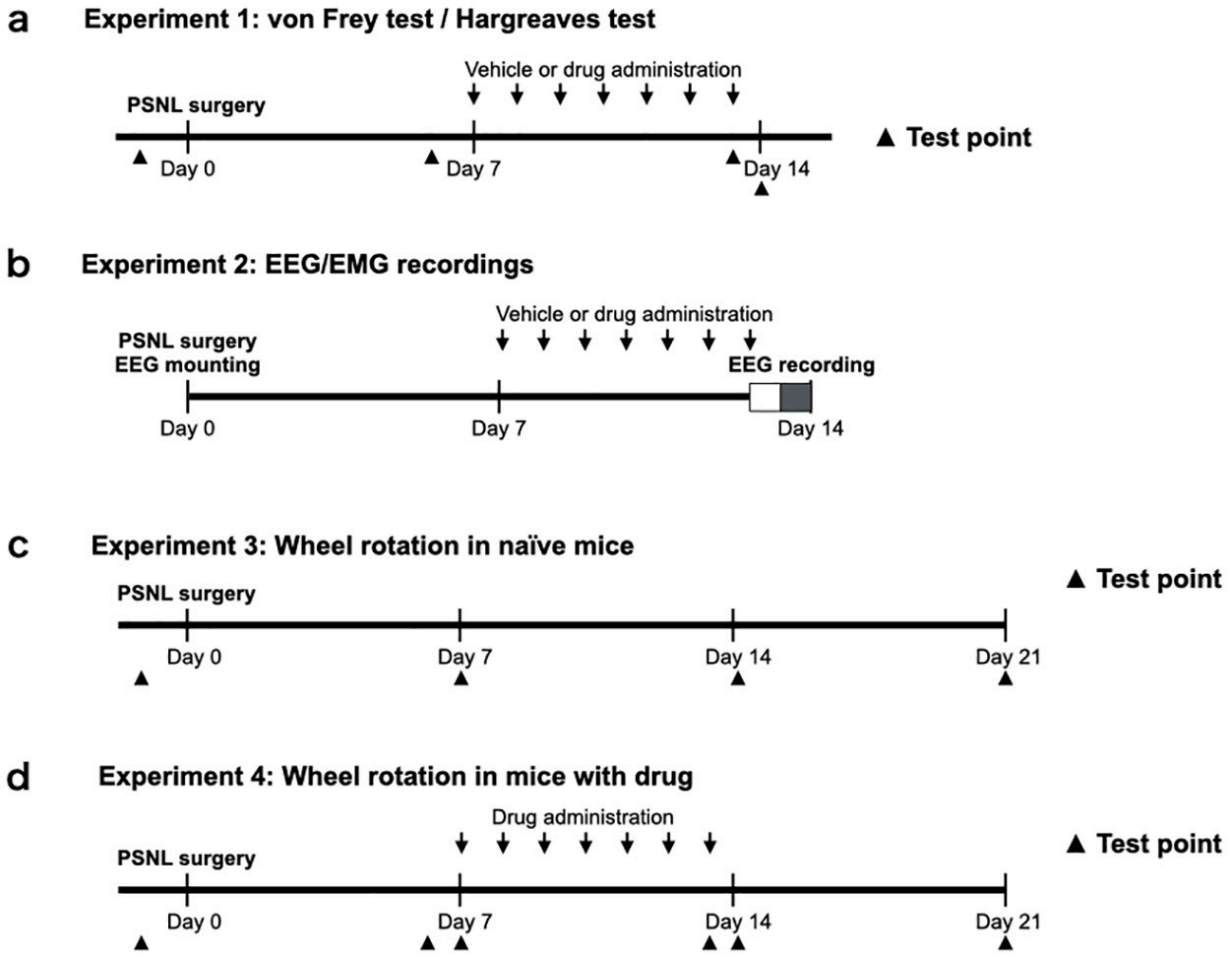
Experimental timeline. Mice were subjected to von Frey and Hargreaves tests at certain test points in Experiment 1 (a). They were treated with suvorexant (30 mg/kg, p.o.), mirtazapine (1 mg/kg, i.p.), or vehicles once daily at ZT 0 for 7 days after nerve ligation. Mice treated with suvorexant or mirtazapine were subjected to EEG for 24 h in Experiment 2 (b). The number of wheel rotations was recorded in naïve mice 1 day before PSNL surgery and at 7, 14, and 21 days after surgery in Experiment 3 (c). The number of wheel rotations was recorded in the mice treated with suvorexant or mirtazapine at the test points in Experiment 4 (d). Abbreviations: ZT, Zeitgeber time; PSNL, partial sciatic nerve ligation; EEG, electroencephalogram.

**Experiment 1**: The von Frey and Hargreaves tests were performed to determine mechanical allodynia and thermal hyperalgesia at certain test points, as shown in Fig 1a. The suvorexant (30 mg/kg) and a control group were administered the drug or the vehicle orally, respectively, for 7 days, 1 week after the PSNL surgery. The mirtazapine (1 mg/kg) and a control group were administered the drug or the vehicle intraperitoneally, respectively, following the same schedule as suvorexant. **Experiment 2**: One week after PSNL and electroencephalogram/electromyogram (EEG/EMG) head-mount implantation surgery, each group was administered suvorexant, mirtazapine, and their respective vehicles for 7 days, then EEG/EMG was measured for 24 h, from 7:00 a.m. on the last day of administration (Fig 1b). The total sleep time, percentage of REM sleep, duration of each parameter, and time change in the power density of the δ wave during non-REM sleep were calculated from EEG/EMG. **Experiment 3**: Mice were habituated in a cage with a rotation wheel for 1 week and then underwent PSNL surgery. The number of wheel rotations was recorded 1 day before surgery and at 7, 14, and 21 days after surgery (Fig 1c). **Experiment 4**: Mice that underwent habituation and PSNL surgery similar to Experiment 3 were treated with suvorexant, mirtazapine, and their respective vehicles from day 7 to day 13 after surgery. The number of wheel rotations was recorded 1 day before surgery and at 6, 7, 13, 14, and 21 days after surgery (Fig 1d).

### Neuropathic pain model

The mice were anesthetized with 3% isoflurane. We produced a PSNL model, as described previously [29]. A tight ligature ligated the right sciatic nerve with an 8–0 silk suture of approximately half of its diameter. In sham-operated mice, nerves were exposed without ligation.

### Compounds and administrations

Suvorexant ([(7R)-4-(5-chloro-1,3-benzoxazol-2-yl)-7-methyl-1,4-diazepan-1-yl][5-methyl-2-(2H-1,2,3-triazol-2-yl)phenyl] methanone) was synthesized as described in previous studies [30, 31]. 30mg/kg suvorexant was dissolved in 0.5% methylcellulose 400 (Wako Pure Chemicals, Japan) immediately before oral administration. Mirtazapine was purchased from. 1mg/kg Mirtazapine (Wako Pure Chemicals, Osaka, Japan) was dissolved in 5% dimethyl sulfoxide and 5% Tween80 before intraperitoneal injection. Methylcellulose and dimethyl sulfoxide were administrated as vehicle controls for suvorexant and mirtazapine, respectively.

### Measurement of mechanical allodynia and thermal hyperalgesia

We performed the von Frey test to assess mechanical allodynia using automated von Frey equipment (Dynamic Plantar Aesthesiometer; Ugo Basile, Italy). The maximum force was set at 5 g to prevent tissue damage, and the ramp speed was 0.25 g/s to achieve an average baseline paw-withdrawal latency of 8–10 s in naive mice [32]. We performed the Hargreaves test to assess thermal hyperalgesia using a radiant heat light source (model 33 Analgesia Meter; IITC/Life Science Instruments, USA). The intensity of the thermal stimulus was adjusted to achieve an average baseline paw-withdrawal latency of 8–10 s in naive mice [33]. The paw-withdrawal latency was determined as the average of four measurements per paw. Only quick hind paw movements (with or without licking of hind paws) away from the stimulus were considered a withdrawal response. Paw movements associated with locomotion or weight shifting were not considered responses. Before these tests, the mice were habituated for at least 20 min in a plastic cage on a metal grid bottom or in a clear acrylic cylinder (15 cm high and 8 cm in diameter) on a glass plate.

### Sleep recordings

We recorded EEG/EMG to evaluate the sleep condition for 24 h on the last day of administration, as previously described [20, 33]. Mice were mounted in a stereotaxic head holder and implanted with EEG and EMG electrodes for polysomnographic recordings (Pinnacle Technology, USA) under 3% isoflurane anesthesia. Two stainless steel EEG recording screws were positioned 1 mm anterior to the bregma or lambda, both 1.5 mm lateral to the midline. EMG activity was monitored using Teflon-coated steel wires placed bilaterally into both trapezius muscles. The collected data were analyzed using appropriate software (SLEEPSIGN Kissei Comtec, Japan). The vigilance of every 10 s epoch was automatically classified into three stages, that is, arousal, rapid eye movement (REM), and non-REM sleep; according to the standard criteria: 1) arousal was defined by a high EMG amplitude and low EEG amplitude; 2) REM sleep was defined by a low EMG amplitude, low EEG amplitude, and high θ wave activity; and 3) non-REM sleep was defined by low EMG amplitude, high EEG amplitude, and high δ wave activity [33]. Defined sleep-wake stages were visually examined, and corrected if necessary. Furthermore, we evaluated the time change in the power density of the δ wave to estimate the quality of sleep. The normalized power density of the δ wave was calculated as a value every 3 h for the total power density of the δ wave during non-REM sleep per day [33].

### Assessment of voluntary activity

Wheel running was measured to assess the voluntary physical performance in a mouse model of neuropathic pain [34]. The mice were allowed to run freely on an open plastic wheel inside a standard cage (Melquest, Japan). Wheel rotations were electronically counted every 24 h and captured in a software program for data storage and analysis at various time points.

### Statistics

**Fig 2:**
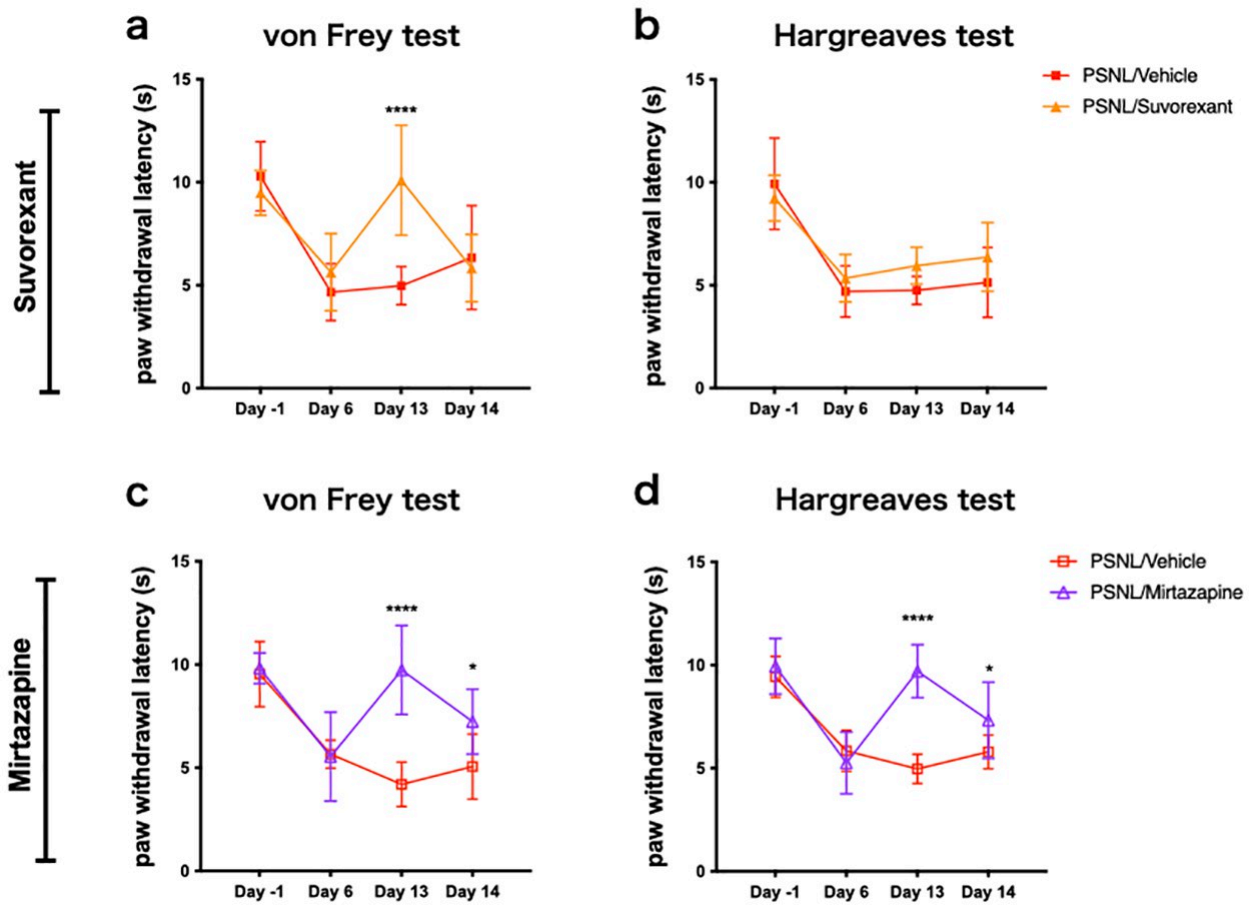
Suvorexant and mirtazapine improved mechanical allodynia and thermal hyperalgesia in the neuropathic pain model. They were assessed for mechanical allodynia (a, c) and thermal hyperalgesia (b, d) with four test points, with the automated von Frey and Hargreaves tests. Data are expressed as means ± SEMs (PSNL-vehicle, n = 8–9; PSNL-suvorexant, n = 8; PSNL-mirtazapine, n = 9). *P < 0.05, ****P < 0.0001, compared by two-way ANOVA with Bonferroni post-test. Abbreviations: ANOVA, analysis of variance; PSNL, partial sciatic nerve ligation; SEM, standard error of the mean; ZT, Zeitgeber time.

**Fig 3:**
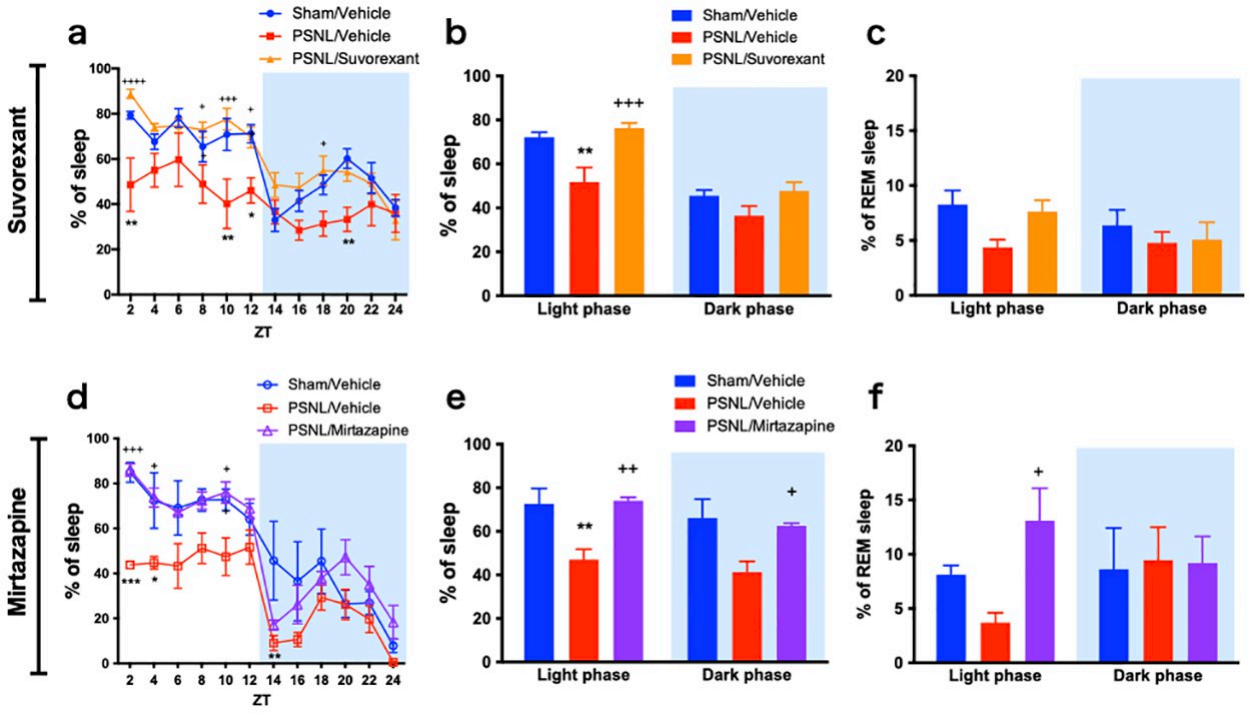
Suvorexant and mirtazapine improved the amount of sleep time in PSNL mice. The time changes of the sleep percentage are shown for every 2 h (a, d) and every 12 h (b, e). The proportions of REM sleep per total sleep were shown (c, f). Data are expressed as means ± SEMs (sham-vehicle, n = 5–6; PSNL-vehicle, n = 5; PSNL-suvorexant, n = 6; PSNL-mirtazapine, n = 5). *P < 0.05, **P < 0.01, and ***P < 0.001 compared between each sham-vehicle and PSNL-vehicle group, and ^+^*P* < 0.05, ^++^*P* < 0.01, ^+++^*P* < 0.001, and ^++++^*P* < 0.0001 compared between each PSNL-vehicle and PSNL-drug (suvorexant or mirtazapine) group by two-way ANOVA with Bonferroni post-test. Abbreviations: ANOVA, analysis of variance; EEG, electroencephalogram; PSNL, partial sciatic nerve ligation; REM, rapid eye movement; SEM, standard error of the mean; ZT, Zeitgeber time.

**Fig 4:**
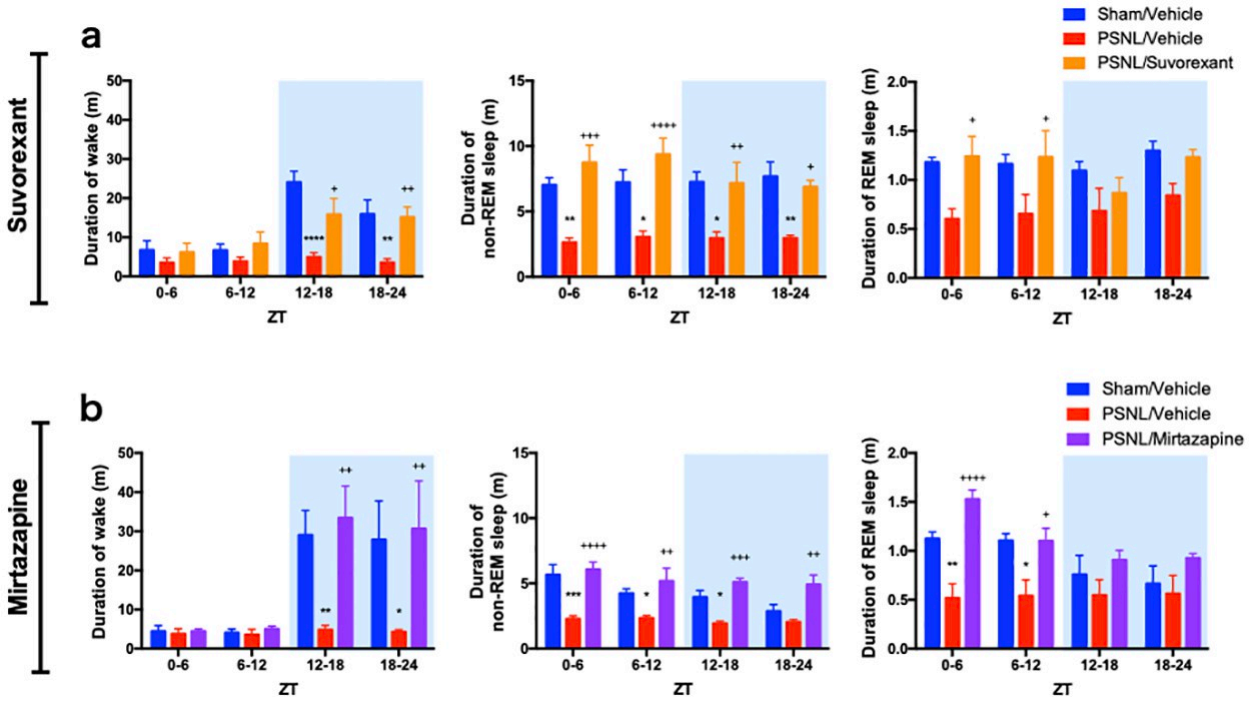
Influence of daily administration of suvorexant or mirtazapine on the duration of arousal and sleep in PSNL. The mean durations of wakefulness, non-REM sleep, and REM sleep in both suvorexant (a) and mirtazapine (b) experiments, were calculated every 6 h. Data are expressed as means ± SEMs (sham-vehicle, n = 5–6; PSNL-vehicle, n = 5; PSNL-suvorexant, n = 6; PSNL-mirtazapine, n = 5). *P < 0.05, **P < 0.01, ***P < 0.001, and ****P < 0.0001 compared between each sham-vehicle and PSNL-vehicle group, and ^+^*P* < 0.05, ^++^*P* < 0.01, ^+++^*P* < 0.001, and ^++++^*P* < 0.0001 compared between each PSNL-vehicle and PSNL-drug (suvorexant or mirtazapine) group by two-way ANOVA with Bonferroni post-test. Abbreviations: ANOVA, analysis of variance; PSNL, partial sciatic nerve ligation; REM, rapid eye movement; SEM, standard error of the mean; ZT, Zeitgeber time.

**Fig 5:**
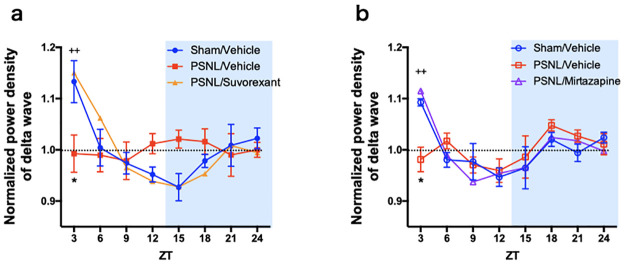
Influence of suvorexant or mirtazapine on the changes in power density of δ wave in PSNL mice. The change in the normalized power density of the δ wave in non-REM sleep was calculated every 3 h. Data are expressed as means ± SEMs (sham-vehicle, n = 5; PSNL-vehicle, n = 4–5; PSNL-suvorexant, n = 5; PSNL-mirtazapine, n = 5). *P < 0.05, compared between each sham-vehicle and PSNL-vehicle group, and ^++^*P* < 0.01, compared between each PSNL-vehicle and PSNL-drug (suvorexant or mirtazapine) groups by two-way ANOVA with Bonferroni post-test. Abbreviations: ANOVA, analysis of variance; PSNL, partial sciatic nerve ligation; REM, rapid eye movement; SEM, standard error of the mean; ZT, Zeitgeber time.

**Fig 6:**
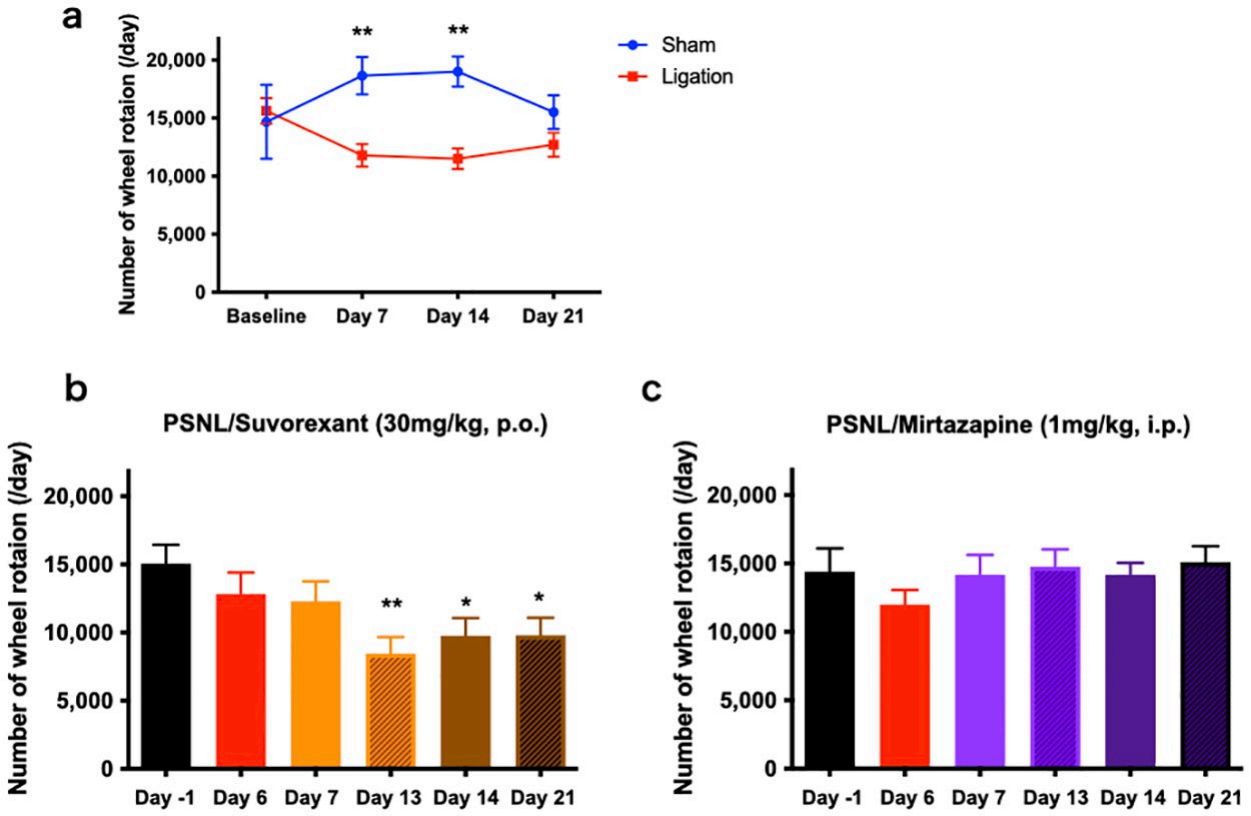
Changes in voluntary activity upon suvorexant and mirtazapine administration following PSNL surgery. The number of rotations in naïve mice was evaluated before and 3 weeks after PSNL surgery and compared with the sham group (a). **P < 0.01, two-way ANOVA with Bonferroni post-test (n = 6). We compared wheel rotations at six different time points with the number in mice treated with each drug at day -1 defined as the baseline (b, c). *P < 0.05, **P < 0.01, compared with baseline by one-way ANOVA with Bonferroni post-test in suvorexant (b) and mirtazapine (d) experiments (n = 8). Data are expressed as means ± SEMs. Abbreviations: ANOVA, analysis of variance; PSNL, partial sciatic nerve ligation; SEM, standard error of the mean; ZT, Zeitgeber time.

All data are expressed as the mean ± standard error of the mean (SEM). Repeated-measures two-way analysis of variance (ANOVA) followed by the Bonferroni multiple comparisons test was used to compare the paw withdrawal latency in von Frey and Hargreaves test (Fig 2), the percentage of each sleep time (Fig 3), the duration of wake, non-REM sleep, and REM sleep time (Fig 4), the normalized power density of δ wave among the “sham-vehicle,” “PSNL-vehicle,” and “PSNL-drug (suvorexant or mirtazapine)” groups on each time point (Fig 5), and the number of wheel rotations between “sham” and “PSNL” groups (Fig 6a). Repeated-measures one-way ANOVA followed by the Bonferroni multiple comparisons test was used to compare the number of wheel rotations at each time point in mice treated with each drug (Fig 6b and 6c). Statistical significance was set at *P* < 0.05. All statistical analyses were performed using Prism version 9.2. (GraphPad Software, La Jolla, CA, USA).

## Results

### Effects of suvorexant and mirtazapine on pain behavior

The paw withdrawal latency in both the von Frey and Hargreaves tests decreased after nerve ligation (day 6) compared with baseline (day -1), as shown in Fig 2a–2d. Suvorexant administration improved the reduction of threshold in the von Frey test (PSNL-vehicle vs. PSNL-suvorexant: 4.98 s vs. 10.1 s, *P* < 0.0001 at day 13; Fig 2a); however, the effect did not last until the next day. There was no significant difference in the response to thermal stimuli (Fig 2b). On the other hand, mirtazapine improved both mechanical allodynia (PSNL-vehicle vs. PSNL-mirtazapine: 4.20 s vs. 9.74 s, *P* < 0.0001 at day 13; Fig 2c) and thermal hyperalgesia (PSNL-vehicle vs. PSNL-mirtazapine: 4.87 s vs. 9.70 s, *P* < 0.0001 at day 13; Fig 2d), these effects persisted until the next day (PSNL-vehicle vs. PSNL-mirtazapine: 5.06 s vs. 7.23 s, *P* = 0.014 (Fig 2c); 5.80 s vs. 7.33 s, *P* = 0.044 (Fig 2d) at day 14). These data suggest that mirtazapine improved PSNL-induced pain behaviors, but the improvement with suvorexant was partial.

### Effects of suvorexant and mirtazapine on the amount of sleep time

We analyzed EEG/EMG in PSNL mice to determine the effects of drugs on the amount of sleep time. We calculated the time change every 2 h of the percentage of sleep (Fig 3a and 3d) and compared the values added up in the light and dark phases (Fig 3b and 3e). The PSNL-vehicle group showed a significant decrease in the percentage of sleep during the light phase, which is the rest period for mice. Suvorexant and mirtazapine significantly normalized the reduction in the percentage of sleep to the same level as the sham group (PSNL-vehicle vs. PSNL-suvorexant: 51.8% vs. 76.3%, P = 0.0007; PSNL-vehicle vs. PSNL-mirtazapine: 47.0% vs. 74.1%, *P* = 0.0050) during the light phase in PSNL (Fig 3b and 3e). In addition, mirtazapine increased the proportion of REM sleep per total sleep (Fig 3f), but not suvorexant (Fig 3c). The proportion of REM sleep was calculated based on the amount of REM sleep and non-REM sleep time (S1 Fig).

### Effects of suvorexant and mirtazapine on the duration time of wakefulness and sleep

Mice are polyphasic sleepers, alternating between sleep and wakefulness in the order of minutes [35]. We calculated the mean duration of wakefulness, non-REM sleep, and REM sleep every 6 hours based on recorded EEG to assess the duration of wakefulness and sleep. The sham mice showed long durations of arousal during the dark phase, which is an active period for mice. However, PSNL mice showed a significant reduction in the duration of wakefulness during the dark phase (PSNL-vehicle vs. sham-vehicle: 4.94 min vs. 24.1 min, *P* < 0.0001 at ZT 12–18; 3.53 min vs. 15.9 min, *P* = 0.0053 at ZT 18–24; Fig 4a, left; and PSNL-vehicle vs. sham-vehicle: 4.83 min vs. 29.0 min, *P* = 0.0098 at ZT 12–18; 4.27 min vs. 27.8 min, *P* = 0.012 at ZT 18–24; Fig 4b, left), which was improved with suvorexant (15.8 min, *P* = 0.012 at ZT 12–18 and 15.2 min, *P* = 0.0063 at ZT 18–24; vs. PSNL-vehicle; Fig 4a, left) and mirtazapine (33.4 min, *P* = 0.0030 at ZT 12–18 and 30.6 min, *P* = 0.0067 at ZT 18–24; vs. PSNL-vehicle; Fig 4b, left). These results suggest that suvorexant and mirtazapine ameliorate the PSNL-induced inability to maintain wakefulness.

Non-REM sleep revealed short duration in the PSNL group at almost all periods, which was improved by suvorexant (PSNL-vehicle vs. PSNL-suvorexant: 2.63 min vs. 8.75 min, P = 0.0001 at ZT 0–6; 3.05 min vs. 9.36 min, *P* < 0.0001 at ZT 6–12; 2.96 min vs. 7.17 min, *P* = 0.0088 at ZT 12–18; 2.96 min vs. 6.88 min, *P* = 0.0162 at ZT 18–24; Fig 4a, middle) and mirtazapine (PSNL-vehicle vs. PSNL-mirtazapine: 2.28 min vs. 6.06 min, *P* < 0.0001 at ZT 0–6; 2.35 min vs. 5.19 min, *P* = 0.0021 at ZT 6–12; 1.94 min vs. 5.11 min, *P* = 0.0005 at ZT 12–18; 2.04 min vs. 4.92 min, *P* = 0.0018 at ZT 18–24; Fig 4b, middle). These results suggest that suvorexant and mirtazapine improved fragmented sleep in PSNL mice.

There was a downward trend or a significant decrease in the duration of REM sleep with nerve PSNL during the light phase. Suvorexant (PSNL-vehicle vs. PSNL-suvorexant: 0.60 min vs. 1.24 min, P = 0.022 at ZT 0–6; 0.65 min vs. 1.23 min, *P* = 0.042 at ZT 6–12; Fig 4a, right) and mirtazapine (PSNL-vehicle vs. PSNL-mirtazapine: 0.52 min vs. 1.53 min, *P* < 0.0001 at ZT 0–6; 0.54 min vs. 1.10 min, *P* = 0.023 at ZT 6–12; Fig 4b, right) significantly increased REM sleep duration in the PSNL group during the light phase.

### Effects of suvorexant and mirtazapine on the normalized power density of δ waves

To assess the change in the depth of sleep, we analyzed the power density of δ waves, which are the factors that define the depth of sleep. The power density of the δ wave during non-REM sleep was calculated every 3 h, and the ratio with the average value during the entire day was calculated for each individual. In the sham group, the power density of the δ wave in non-REM sleep was particularly strong during the early light phase. The δ power tended to weaken from the late light phase to the early dark phase and became stronger again toward the latter half of the dark phase. However, in the PSNL-vehicle group, diurnal fluctuation of δ power became ambiguous; in particular, the power density of the δ wave in the early light phase was significantly reduced (PSNL-vehicle vs. sham-vehicle: 0.99 vs. 1.13, *P* = 0.020 at ZT 0; Fig 5a; and PSNL-vehicle vs. sham-vehicle: 0.98 vs. 1.09, *P* = 0.0042 at ZT 0; Fig 5b). Both suvorexant (1.15, *P* = 0.0074; vs. PSNL-vehicle; Fig 5a) and mirtazapine (1.12, *P* = 0.0004; vs. PSNL-vehicle; Fig 5b) improved the reduction in δ power density. These data suggest that administration of suvorexant and mirtazapine attenuated PSNL-induced changes in the depth of sleep.

### Effects of suvorexant and mirtazapine on the voluntary activity

To assess voluntary activity, we placed the mice in cages with a running wheel and recorded the number of wheel rotations. PSNL significantly reduced the wheel rotation compared to sham (sham vs. PSNL: 18,650/day vs. 11,801/day, *P* = 0.0040 at day 7; 19,010/day vs. 11,500/day, *P* = 0.0015 at day 17; Fig 6a). Suvorexant and mirtazapine were administered after PSNL surgery, and the wheel rotation was evaluated at certain test points, as shown in Fig 6b. Suvorexant treatment reduced the number of wheel rotations compared with the baseline. On the other hand, mirtazapine treatment did not change the number of wheel rotations (Fig 6c). These results suggest that mirtazapine improves voluntary activity in PSNL mice.

## Discussion

Recent clinical and basic research suggest that sleep disorder is a heterogenic disorder and requires multifaceted evaluation to correctly determine the state of sleep and the effects of therapeutic agents [36–38]. We evaluated the sleep status of a preclinical pain model to be close to clinical situations by examining the amount of sleep time, sleep duration, sleep depth, and the effect on daytime activity objectively using EEG or wheel rotation. PSNL caused a decrease in REM and non-REM sleep time based on EEG analysis (Fig 3). In addition, we found that the duration of non-REM sleep was interrupted, resulting in fragmented sleep in the neuropathic pain model (Fig 4). The duration of wakefulness during the dark phase was substantially reduced in the neuropathic pain model mice compared with that in the sham group (Fig 4). Non-REM sleep is classified into stages based on depth, which correlates with the strength of the δ power density. The sham group showed deep sleep in the early stages of the light phase with a high power density of δ wave, following which the δ power gradually became shallower towards the late stages of the light phase. In contrast, such early deep sleep was not observed in the PSNL vehicle group (Fig 5). Furthermore, PSNL reduced the number of wheel rotations (Fig 6), indicating that sleep was shortened and fragmented, the rhythm of sleep depth was lost, and arousal and voluntary activity were impaired due to chronic pain caused by nerve injury.

The orexin receptors (orexin-1 and orexin-2 receptor) are involved in stabilizing arousal and suppressing sleep by maintaining the activity of monoaminergic neurons in the central arousal system [21, 22]. Suvorexant could be a potential therapeutic agent to improve pain-related sleep disorders because it causes pharmacological inhibition of orexin receptors [23, 24]. In the present study, suvorexant improved chronic pain-related changes in sleep parameters. Suvorexant improved sleep time by increasing non-REM and REM sleep in the chronic neuropathic pain model (Fig 3). Suvorexant also improved the fragmentation of non-REM sleep, non-sustainability of arousal (Fig 4), and loss of rhythm in sleep depth (Fig 5). However, suvorexant did not improve the voluntary activity (Fig 6). Orexin can potentiate the excitatory synaptic transmission of dopaminergic neurons in the mesolimbic system, causing motivation [39]. Furthermore, the mesopontine tegmentum, including structures relevant to locomotion and muscle tone, is also a major target of orexin [39]. These findings suggest that blocking orexin signaling may lead to lower locomotive behavior.

Several studies have shown that mirtazapine, which is commonly used clinically, may improve sleep disorders [26, 27, 40, 41]. The sedative effects of mirtazapine are thought to be mediated by suppressing histaminergic and serotonergic neurons in the central arousal system because mirtazapine shows strong antagonistic effects on the H_1_ histamine receptor and 5HT_2_ serotonin receptor [27]. The effects of mirtazapine on the endocrine system by normalizing the hypothalamo-pituitary-adrenocortical system overactivation and blunting the melatonin system could be a potential mechanism for sleep improvement [41]. A previous study showed that 1 mg/kg of mirtazapine improves only sleep quantity in PSNL mice, but no other symptoms have been observed [42]. Analysis of multiple parameters of sleep revealed that mirtazapine improved the reduction in sleep time, sleep fragmentation, and depth of sleep onset. The results of the wheel rotation experiment showed an improving trend in the decline of involuntary movement when PSNL mice were treated with mirtazapine. Mirtazapine was reported to promote active motion and have anti-immobility effects in a rat forced swimming test [43]. The enhancing effects of mirtazapine on motivation may be related to increased dopamine release in the frontal cortex [44, 45]. However, in a clinical trial, up to 54% of patients reported daytime sleepiness as an adverse effect of mirtazapine [40]. Mirtazapine predominantly produces anti-histaminergic effects at lower doses, whereas noradrenergic effects become more predominant at higher doses [46]. Due to this unique pharmacological profile, mirtazapine is thought to produce relatively more sedation at lower doses.

In this study, mirtazapine significantly increased the REM sleep time in PSNL. The effect of the amount of REM sleep on animal health remains unclear, and further research is needed. A recent study on REM sleep and mortality showed that for every 5% decrease in REM sleep, mortality increased by 13% [47]. Strategies to preserve REM sleep may reduce the mortality risk in patients with reduced REM sleep. On the other hand, some antidepressants that increase REM sleep are associated with nightmares and REM sleep behavior disorder as side effects [48] because nightmares are essentially a REM pathology [49]. However, there is no consistent view on the effect of mirtazapine on REM sleep, with both a report that mirtazapine increases REM sleep [50] and that it does not change [51].

We investigated the effects of suvorexant and mirtazapine on chronic pain-related changes in sleep parameters and voluntary physical performance in a preclinical model. However, the specific neurological mechanisms by which these agents regulate chronic pain-related sleep disorders require further investigation.

## Conclusions

We investigated the influence of suvorexant and mirtazapine on pain-related changes in sleep parameters. We found that suvorexant and mirtazapine could be potential therapeutic agents for pain-related sleep disorders, but further studies are needed. These drugs showed different pharmacological profiles in REM sleep, pain behavior, and voluntary activity. These findings may be helpful for understanding the pharmacological properties of each sleep drug and avoid undesirable side effects.

## Supporting information

S1 Fig Influence of daily administration of suvorexant and mirtazapine on non-REM and REM sleep in the neuropathic pain model. The amount of non-REM sleep and REM sleep time were shown every 2 hrs (a, c) or every 12 hrs (b, d). Data were expressed as the mean ± SEM (sham-vehicle, n = 5–6; PSNL-vehicle, n = 5; PSNL-suvorexant, n = 6; PSNL-mirtazapine, n = 5). *P < 0.05, **P < 0.01, and ***P < 0.001 compared between each sham-vehicle and PSNL-vehicle group, and +P < 0.05, ++P < 0.01, +++P < 0.001, and ++++P < 0.0001 compared between each PSNL-vehicle and PSNL-drug (suvorexant or mirtazapine) group by two-way ANOVA with Bonferroni post-test. Abbreviations: PSNL, partial sciatic nerve ligation; EEG, electroencephalogram; REM, rapid eye movement; ZT, zeitgeber time.
(TIF)
