# Supplementary figures and images for: Suvorexant and mirtazapine improve chronic pain-related changes in parameters of sleep and voluntary physical performance in mice with sciatic nerve ligation
Source: PLoS One. 2022 Feb 25;17(2):e0264386. doi: 10.1371/journal.pone.0264386 (PMC8880854; doi:10.1371/journal.pone.0264386)

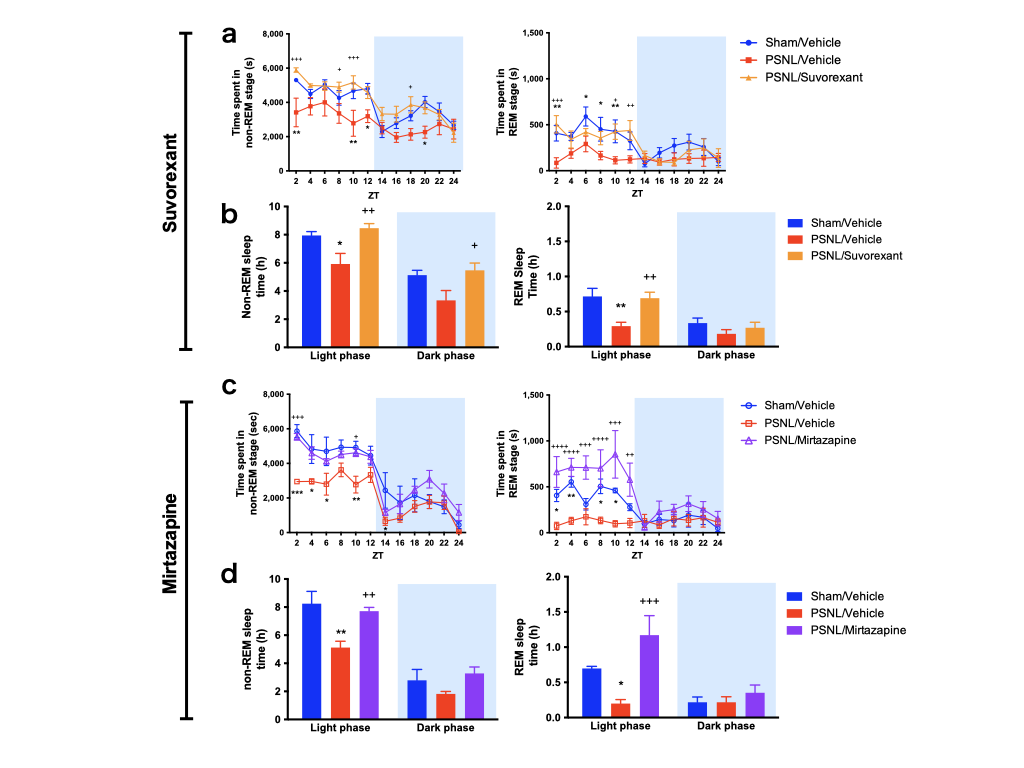

Supplement: S1 Fig — The amount of non-REM sleep and REM sleep time were shown every 2 hrs (a, c) or every 12 hrs (b, d). Data were expressed as the mean ± SEM (sham-vehicle, n = 5–6; PSNL-vehicle, n = 5; PSNL-suvorexant, n = 6; PSNL-mirtazapine, n = 5). *P < 0.05, **P < 0.01, and ***P < 0.001 compared between each sham-vehicle and PSNL-vehicle group, and +P < 0.05, ++P < 0.01, +++P < 0.001, and ++++P < 0.0001 compared between each PSNL-vehicle and PSNL-drug (suvorexant or mirtazapine) group by two-way ANOVA with Bonferroni post-test. Abbreviations: PSNL, partial sciatic nerve ligation; EEG, electroencephalogram; REM, rapid eye movement; ZT, zeitgeber time. (TIF) [file pone.0264386.s001.tif]
